# Analysis of the impact of the trade war between China and America on the RMB exchange rate under the R-vine copula model from the perspective of the global value chain

**DOI:** 10.1007/s10660-023-09680-x

**Published:** 2023-03-31

**Authors:** Chao Yang, Wee-Yeap Lau

**Affiliations:** Faculty of Business and Economics, University of Malaya, 50603 Kuala Lumpur, Malaysia

**Keywords:** Global value chain, R-vine copula model, Trade war between China and America, RMB exchange rate, Upper and lower tail dependency coefficient

## Abstract

Changes in the RMB exchange rate (ExRate) impact major strategic directions, such as China’s rise, the “Belt and Road” initiative, and RMB internationalization. Studying the impact of the trade war between China and America on the RMB ExRate is significant. This paper constructs an R-vine copula model and studies the influencing factors of the ExRate and the mechanism of the trade war between China and America on the RMB ExRate. The impact of the Trade war between China and America on the RMB ExRate is analyzed in detail. Besides, the R-vine copula model is used to analyze the fluctuations of the ExRate returns of the Association of Southeast Asian Nations (ASEAN). The R-vine copula model is employed to study the volatility of the ExRate return series of ASEAN countries, and the mean of the ExRate dependence coefficients of ASEAN and the estimation results of R-vine copula parameters are analyzed. Finally, the results are obtained. There is a relation between the RMB ExRate and Chinese tariffs in analyzing the impact of the trade war between China and America on the RMB ExRate. There is a negative correlation between tariffs and the RMB ExRate. There is a positive correlation between the value of the RMB and China’s trade surplus without considering the monetary policy. The value of the Chinese currency has risen as China’s trade surplus has grown. Stable foreign exchange reserves can stabilize the RMB ExRate. It is concluded that the economic and trade cooperation between China and ASEAN has become increasingly frequent under the influence of the trade war between China and America through the R-vine copula model analysis. The ExRate risk further increases and the ExRate yield sequence fluctuates significantly. The lower tail dependency coefficient for market tail dependencies is slightly larger than the upper tail dependency coefficient. The impact of bad news on the currency ExRate market is clear. In the context of the current trade war between China and America intensifying, the sharp fluctuations in the RMB ExRate caused by the trade war between China and America have promoted the transformation of economic growth. The shift from commodity export to capital export is realized. The reasons for RMB ExRate changes need to be explored. It is recommended to continuously deepen the reform of the RMB ExRate system and maintain the stable development of the ExRate.

## Introduction

Global economic integration and the emergence of the information age have given birth to new production and trade models such as international production segmentation, procurement, outsourcing, and intra-company trade. It gives birth to the rapid development of the global value chain to form a new system of international production and trade [1]. The adjustment of the RMB exchange rate (ExRate) will change China’s foreign trade development level and economic development level. In addition, it will also impact major strategic policies such as China’s rise, the “Belt and Road” initiative, and RMB internationalization. Therefore, it is very important to study the influence of the trade war between China and America on the RMB ExRate [2].

Henderson and Hooper (2021) constructed instrumental variables based on the policy documents on the encouragement of technology introduction implemented by the Chinese government to study the impact of the trade war between China and America on the US technology blockade against China on the accumulation of high-level scientific research talents in Chinese manufacturing enterprises [3]. The important time points of the trade war between China and America conflict were selected as the time window to study the relationship between the conflict between the two countries and the Chinese stock market [4]. Qiu et al. (2019) analyzed the real reasons for the trade war between China and America, objectively evaluated the spillover effect of the China-US trade war on China's reform and opening up from a historical perspective, and finally proposed rational strategies to deal with the China-US trade war [5]. Li (2022) studied short-term fluctuation rules of the RMB ExRate by building an Autoregressive moving average model (ARIMA) and predicted the fluctuation trend of the RMB ExRate against the USD according to the ARIMA model [6]. When the input sequence {u(n)} and output sequence {a(n)} of the ARIMA model can both be measured, the least square method can be employed to estimate the model parameters. Such estimation is a linear estimation, and the model parameters can be estimated with sufficient precision. In the actual analysis of financial time series, the ARIMA model is used as a regression function to fit the original sequence. The ARIMA model can be used for the return on assets to eliminate the autocorrelation of assets [7]. Liang (2022) studied the impact of Sino-US trade friction on RMB ExRate fluctuations based on the Exponential Generalized Auto Regressive Conditional Heteroskedasticity (EGARCH) model [8]. Arreola Hernandez et al. (2017) used r-vine, c-vine, and d-vine theories to study the dependency risk characteristics of 20 stock portfolios of Australian retail, manufacturing, and gold mining equity sectors before, during, and after the 2008–2009 global financial crisis [9]. Nagler et al. (2022) proposed computationally efficient methods for estimation, simulation, prediction, and uncertainty quantification using the Vine copula graph model, and proved their effectiveness through asymptotic results and simulation [10]. Zhang et al. (2022) paid attention to the direct and indirect systemic risk spillovers among East Asian, European, and American stock markets during the COVID-19 epidemic. On account of the Copula CoVaR model, the direct spillover matrix of systemic risk was constructed, and the indirect spillover path through R-vine was further explored [11]. Through literature analysis, it is found that there are not many studies on the impact of the trade war between China and America on the RMB ExRate from the perspective of the global value chain. The research and analysis of the relevant structure of the financial market not only have important theoretical and practical significance for risk measurement but also help to improve the scientific nature and accuracy of financial decisions. The Vine copula model does not have dimensional limitations. It has high flexibility and accuracy in describing the correlation structure between high-dimensional portfolio assets, which helps investors to construct portfolios reasonably and reduce risks. The C-vine copula model and D-vine copula model are widely used, while there are few pieces of literature that use the R-vine copula model to solve problems. This is mainly due to the flexible structure of the R-vine, which leads to certain difficulties in practical operation. In recent years, due to the development of computers, the operation of the R-Vine copula model has become simpler, thus increasing the application of the R-vine copula model in the financial field [12]. For example, Peng and Ke (2022) used the R-vine copula model to study the relationship between the risk spillover effect between the real estate industry and the financial industry, analyze the dependent structure between industries and the risk spillover path, and measure the risk spillover effect between industries [13].

This paper studies the mechanism of the trade war between China and America on the RMB ExRate from the global value chain perspective. The RMB ExRate market is analyzed by constructing the R-vine copula model. The impact of the trade war between China and America on the RMB ExRate is focused on. The RMB ExRate fluctuates wildly during the trade war between China and America. The impact of the trade war between China and America on the RMB ExRate is studied by creating an ExRate determination model. This can scientifically reflect the impact of the trade war on the RMB ExRate and the core factors of ExRate changes. As a result, corresponding policies are provided for the Chinese government to deal with the adverse economic impact of the trade war.

## Theoretical basis and method of research

### Definition of global value chains

**Fig. 1:**
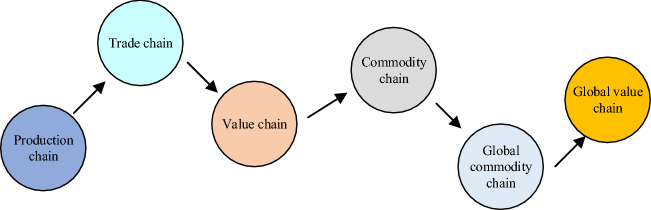
Evolution of global value chains

In the 1970s, the theory of trade in intermediate goods could be considered the earliest understanding of global value chains. At that time, there was a global trade division beyond national boundaries, but the production process was too small-scale, and the production process could be divided according to each country's advantages in the production process [14]. The formation of a commodity’s value needs to be completed by the value-creation behaviors of multiple production links in different countries or regions under this division of labor mode. A complete set of processes for a commodity is divided into a vertical production chain. The global value chain is the formation process of a product in multiple links in domestic and foreign markets, mainly composed of several stages such as research, development, design, raw material procurement, configuration, production, assembly, marketing, and logistics [15]. Figure 1 shows its evolution.

In Fig. 1, the definition of the global value chain has evolved from the initial production chain, trade chain, and value chain, gradually to the commodity chain and global commodity chain, and finally to the current global value chain. A vertical production chain is a process from beginning to end. The global value chain embodies all the value processes in the process from scratch to final consumption and scrap of a product under the international division of labor system. Supply chains can be divided into vertical and horizontal. In the horizontal supply chain, independent suppliers specialize in their business field and create unique competitiveness in the fierce competition environment, which can provide customers with more cost-effective products or services. The supply chain went from vertical to horizontal and back again, creating a spiral upward. The commodity chain is an internal organization of the network system, with the particularity of the location, social and local combination. The analysis framework of the global commodity chain portrays the complexity of globalized production to some extent but does not touch on the exploitative nature and labor relations behind it. The types of global commodity chains include two management structures: producer-driven and buyer-driven.

The foreign exchange market faces obvious political risks, whether it is policy change, government intervention, war and turmoil, nationalization risk, terrorist activities, cyber threats, etc., all of which may threaten the stability of the ExRate market. Such political risks are sudden, so they often exceed the normal expectations of investors and the market, resulting in a large and rapid rise or fall of spot prices in the foreign exchange market in a short period of time. The conclusion of economic and trade agreements of the first phase of China-US trade relations is a milestone since the outbreak of the trade dispute. The agreement covers a wide range of areas, mainly focusing on China's intellectual property protection (IPR), technology transfer, food and agricultural products, service market access, ExRate as well as trade expansion, and has also established a bilateral dispute settlement mechanism. This is a good start. Both China and the United States have chosen the practical option of pledging their obligations and reducing trade conflicts, reversing the downward spiral in China-US trade relations. The marginal distribution of the Copula function can accurately describe the correlation structure of the assets of each enterprise in the global value chain.

### R-vine copula model

#### Copula function theory

The Copula function is employed to connect the return rate series of industries described by edge distribution. It is a joint probability distribution function proposed by Sklar that follows uniform distribution at [0,1] and can be used to describe the correlation between combination variables [16]. Copula functions are very important for the modeling of risk assets in financial markets. Because they not only can accurately depict the relevant structure among assets, but also help investors to flexibly construct multiple distributions to adapt to financial assets. When describing the dependence relationship between financial markets, the Copula function can accurately describe the characteristics such as nonlinearity and heavy tail, so it has advantages beyond the reach of traditional methods.

In Sklar’s theorem, an $n$-dimensional cumulative distribution function $E$ is given. Each edge distribution is ${E}_{1},{E}_{2},\cdots {E}_{n}$. Then there is a copula function $C$.

$E\left({x}_{1},{x}_{2},{x}_{3}\cdots {x}_{n}\right)$ is the joint distribution function of *n*-dimensional random variables $X=\left({X}_{1},{X}_{2},{X}_{3}\cdots {X}_{n}\right)$. ${E}_{i}$ is the marginal distribution function of the random variable. The multivariate distribution function expressed by the Copula function is:

1 $$E\left({x}_{1},{x}_{2},{x}_{3}\cdots {x}_{n}\right)=B\left({E}_{1}\left({x}_{1}\right),{E}_{2}\left({x}_{2}\right),\cdots {E}_{n}\left({x}_{n}\right)\right)$$

In Eq. (1), *C* is an existing Copula function. ${E}_{1}\left({x}_{1}\right),{E}_{2}\left({x}_{2}\right),\cdots {E}_{n}\left({x}_{n}\right)$ are marginal distribution functions.

If all marginal distributions ${E}_{1}\left({x}_{1}\right),{E}_{2}\left({x}_{2}\right),\cdots {E}_{n}\left({x}_{n}\right)$ are continuous, then Copula function $C\left({x}_{1},{x}_{2},{x}_{3}\cdots {x}_{n}\right)$ is uniquely determined, where $\left({u}_{1},{u}_{2},{u}_{3}\cdots {u}_{n}\right)$ obeys the upper uniform distribution of [0,1].

2 $$C\left({u}_{1},{u}_{2},{u}_{3}\cdots {u}_{n}\right)=E({{E}_{1}}^{-1}\left({u}_{1}\right),{{E}_{2}}^{-1}\left({u}_{2}\right),\cdots {{E}_{n}}^{-1}\left({u}_{\mathrm{n}}\right))$$

In Eq. (2), $${u}_{1}={E}_{1}\left({x}_{1}\right)$$, $${u}_{2}={E}_{2}\left({x}_{2}\right)$$, $${u}_{3}={E}_{3}\left({x}_{3}\right)$$.

Equation (3) shows the density function of the multivariate distribution function.

3 $$f\left({x}_{1},{x}_{2}\cdots {x}_{n}\right)=\mathrm{c}\left({E}_{1}\left({x}_{1}\right),{E}_{2}\left({x}_{2}\right),\cdots {E}_{n}\left({x}_{n}\right)\right)\cdot {f}_{1}\left({x}_{1}\right)\cdots {f}_{n}\left({x}_{n}\right)$$

In Eq. (3), the edge density function is ${f}_{i}\left({x}_{i}\right)$, and the *n*-dimensional copula density function is *c*.

There are three kinds of common bivariate Copula functions. The first kind is the most basic elliptic Copula. Typical functions are Gaussian Copula and T-Copula; The second kind is Archimedean Copula, including Clayton Copula, symmetric Frank Copula, etc., which is sensitive to the lower tail; The third kind is extreme value Copula, such as Gumble Copula, which is sensitive to the upper tail [17].

#### Correlation measure of Copula function

Kendall rank correlation coefficient can be used to measure the consistency of the variation trend of random variables, and the correlation coefficient between the two variables can be examined by observing whether the variation trend between the two variables is consistent. If the trend is consistent, it indicates that there is a positive correlation between the two variables. Otherwise, it is a negative correlation. It can describe not only the linear correlation of the n-dimensional cumulative distribution function but also depict the nonlinear correlation. The Kendall rank correlation coefficient $\sigma$ is the degree of consistency between the changes of two variables [12]. It is expressed as follows. Suppose $\left({x}_{1},{y}_{1}\right)$ and $\left({x}_{2},{y}_{2}\right)$ are independent and identically distributed vectors, ${x}_{1},{x}_{2}\in x$, ${y}_{1},{y}_{2}\in y$, then, $\sigma$ is expressed as:

4 $$\sigma =Q\left\{\left({x}_{1}-{x}_{2}\right)\left({y}_{1}-{y}_{2}\right)>0\right\}-Q\left\{\left({x}_{1}-{x}_{2}\right)\left({y}_{1}-{y}_{2}\right)<0\right\}$$

Equation (5) can be obtained by the simple transformation.

5 $$\sigma =2Q\left\{\left({x}_{1}-{x}_{2}\right)\left({y}_{1}-{y}_{2}\right)>0\right\}-1$$

Assuming that the Copula function corresponding to $\left(x,y\right)$ is $D(u,v)$, $\sigma$ can be given by the corresponding Copula function, as shown in Eq. (6).

6 $$\sigma =4{\int }_{0}^{1} {\int }_{0}^{1} B(u,v)dB(u,v)-1$$

The upper and lower tail dependence coefficient represents the conditional probability that when a given random variable exceeds the value of a specific quantile function at a specified confidence level, another random variable exceeds the value of a specific quantile function at the corresponding confidence level.

Under extreme value theory, the upper and lower tail dependence coefficient can measure the dependence of two variables under extreme conditions (price boom or crash). By analyzing the tail correlation between financial markets through the upper and lower tail dependence coefficient of the Copula function, the mutual influence relationship between two markets under extreme circumstances can be studied more intuitively.

It can be assumed that the distribution function of random variables *X*, *Y* are *E*, *H*. the corresponding copulas function is *B*. *X* and *Y* are the upper and lower tail dependence coefficient based on the copula function B. ${\lambda }_{U}$ and ${\lambda }_{L}$ are shown in Eqs. (7) and (8):

7 $${\lambda }_{U}(\alpha )=\underset{\alpha \uparrow 1}{lim} P\left(Y>{H}^{-1}(\alpha )\mid X>{E}^{-1}(\alpha )\right)=\underset{\alpha \uparrow 1}{lim} \frac{1-2\alpha +B(\alpha ,\alpha )}{1-\alpha }$$

8 $${\lambda }_{L}(\alpha )=\underset{\alpha \downarrow 0}{lim} Q\left(Y<{H}^{-1}(\alpha )\mid X<{E}^{-1}(\alpha )\right)=\underset{\alpha \downarrow 0}{lim} \frac{B(\alpha ,\alpha )}{\alpha }$$

In Eqs. (7)–(8), $\alpha$ represents the confidence level.

#### R-vine copula theory

Bivariate copula strategy Vine copula is a multivariate copula that connects the Pair-Copula together through vine structure. Various vine structures consider the dependency relationship among asset variables through the certain regular tree structure. Compared with other copulas, the Vine copula is more flexible in describing the dependencies between high-dimensional data and complex variables. The Vine copula model is usually used to capture the dependencies between high-dimensional complex variables [11, 18]. The construction of the Vine copula is mainly implemented in Pair-copula constructions (PCCs), which decompose the multivariate copula into the product of conditional and non-conditional Pair-copula. And the choice of each Pair-copula is independent of the other. Therefore, the Vine copula model is superior to multivariate time series.

In Sklar’s theorem, ${E}_{X\mid W}(\cdot \mid w),{E}_{Y\mid W}(\cdot \mid w)$ are conditional distributions of $X|W=w,Y|W=w$ respectively; ${E}_{X\mid W}(\cdot \mid w)$) is the joint conditional distribution of $(X,Y)\mid W=w$; the support set of *W* is *M*, and it is assumed that for all $w \in M$, $F_{{X\left| W \right.}} \left( { \cdot w} \right)\;{\mkern 1mu} {\text{and}}\;F_{{Y\left| W \right.}} \left( { \cdot \left| {\text{w}} \right.} \right)$ is continuous for both *x* and *y* respectively, then there is a unique condition copula $B(\cdot \mid w)$, as follows:

9 $${E}_{XY\mid W}(x,y\mid w)=C\left({E}_{X\mid w}(x\mid w),{E}_{Y\mid W}(y\mid w)w\right)\forall (x,y)\in \overline{R }\times \overline{R },w\in M$$

On the contrary, if ${E}_{X\mid W}(\cdot \mid w),{E}_{Y\mid W}(\cdot \mid w)$ are the conditional distribution of $X|W=w,Y|W=w$ respectively; $\{c(\cdot \mid w)\}$ is the conditional copula family of *w* metric, then the function ${F}_{XY\mid W}(\cdot \mid w)$ is defined by Eq. (7) that is the conditional bivariate distribution function under the conditional marginal distribution. ${E}_{X\mid W}(\cdot \mid w),{E}_{Y\mid W}(\cdot \mid w)$ are the conditional bivariate distribution function.

On the basis of the conditional Sklar's theorem, in the same way as Sklar's theorem, the following conditional density function can be obtained by taking partial derivatives, as illustrated in Eq. (10).

10 $$\begin{gathered} f_{{XY|W}} \left( {x,y|w} \right) \equiv \frac{{\partial ^{2} E_{{XY|W}} \left( {x,y|w} \right)}}{{\partial x\partial y}} \hfill \\ = \frac{{\partial ^{2} C\left( {E_{{X|W}} \left( {x|w} \right),E_{{Y|W}} \left( {y|w} \right)w} \right)}}{{\partial E_{{X|W}} \left( {x|w} \right)\partial E_{{Y|W}} \left( {y|w} \right)}} \cdot \frac{{\partial E_{{X|W}} \left( {x|w} \right)}}{{\partial x}} \cdot \frac{{\partial E_{{Y|W}} \left( {y|w} \right)}}{{\partial y}} \hfill \\ = f_{{X|W}} \left( {x|w} \right) \cdot f_{{Y|W}} \left( {y|w} \right) \cdot c\left( {E_{{X|W}} \left( {x|w} \right),E_{{Y|W}} \left( {y|w} \right)w} \right) \hfill \\ \forall \left( {x,y,w} \right) \in \bar{R} \times \bar{R} \times M \hfill \\ \end{gathered}$$

If ${X=\left({X}_{1},{X}_{2},{X}_{3}\right)}^{\mathrm{^{\prime}}}\sim E$, the decomposed result of the joint density function f of *X* can be obtained according to the knowledge of probability theory, as demonstrated in Eq. (11).

11 $$f\left({x}_{1},{x}_{2},{x}_{3}\right)={f}_{1}\left({x}_{1}\right){f}_{2\mid 1}\left({x}_{2}\mid {x}_{1}\right){f}_{3\mid \mathrm{1,2}}\left({x}_{3}\mid {x}_{1},{x}_{2}\right){\left({x}_{1},{x}_{2},{x}_{3}\right)}^{\mathrm{^{\prime}}}\in {R}^{3}$$

${f}_{2\mid 1}\left({x}_{2}\mid {x}_{1}\right)$ can be obtained according to the conditional density function and Sklar’s theorem, as shown in Eq. (12).

12 $$\begin{array}{c}{f}_{2\mid 1}\left({x}_{2}\mid {x}_{1}\right)=\frac{{f}_{\mathrm{1,2}}\left({x}_{1},{x}_{2}\right)}{{f}_{1}\left({x}_{1}\right)}=\frac{{c}_{\mathrm{1,2}}\left({F}_{1}\left({x}_{1}\right),{E}_{2}\left({x}_{2}\right)\right){f}_{1}\left({x}_{1}\right){f}_{2}\left({x}_{2}\right)}{{f}_{1}\left({x}_{1}\right)}\\ ={c}_{\mathrm{1,2}}\left({E}_{1}\left({x}_{1}\right),{E}_{2}\left({x}_{2}\right)\right){f}_{2}\left({x}_{2}\right)\end{array}$$

In Eq. (12), ${c}_{\mathrm{1,2}}$ represents a pair-copula between $\left({E}_{1}\left({x}_{1}\right) and {E}_{2}\left({x}_{2}\right)\right)$. The same can be obtained according to Sklar’s theorem and the conditional density function.

13 $$\begin{array}{c}{f}_{3\mid \mathrm{1,2}}\left({x}_{3}\mid {x}_{1},{x}_{2}\right)=\frac{{f}_{\mathrm{2,3}\mid 1}\left({x}_{2},{x}_{3}\mid {x}_{1}\right)}{{f}_{2\mid 1}\left({x}_{2}\mid {x}_{1}\right)}\\ =\frac{{c}_{\mathrm{2,3}\mid 1}\left({E}_{2\mid 1}\left({x}_{2}\mid {x}_{1}\right),{F}_{3\mid 1}\left({x}_{3}\mid {x}_{1}\right)\mid {x}_{1}\right){f}_{2\mid 1}\left({x}_{2}\mid {x}_{1}\right){f}_{3\mid 1}\left({x}_{3}\mid {x}_{1}\right)}{{f}_{2\mid 1}\left({x}_{2}\mid {x}_{1}\right)}\\ ={c}_{\mathrm{2,3}\mid 1}\left({E}_{2\mid 1}\left({x}_{2}\mid {x}_{1}\right),{E}_{3\mid 1}\left({x}_{3}\mid {x}_{1}\right)\mid {x}_{1}\right){f}_{3\mid 1}\left({x}_{3}\mid {x}_{1}\right)\\ ={c}_{\mathrm{2,3}\mid 1}\left({E}_{2\mid 1}\left({x}_{2}\mid {x}_{1}\right),{E}_{3\mid 1}\left({x}_{3}\mid {x}_{1}\right)\mid {x}_{1}\right){c}_{\mathrm{1,3}}\left({E}_{1}\left({x}_{1}\right),{E}_{3}\left({x}_{3}\right)\right){f}_{3}\left({x}_{3}\right)\end{array}$$

Therefore, the joint density function can be obtained.

14 $$\begin{array}{c}f\left({x}_{1},{x}_{2},{x}_{3}\right)={c}_{\mathrm{2,3}\mid 1}\left(E\left({x}_{2}\mid {x}_{1}\right),E\left({x}_{3}\mid {x}_{1}\right)\right){c}_{\mathrm{1,3}}\left({E}_{1}\left({x}_{1}\right),{E}_{3}\left({x}_{3}\right)\right){c}_{\mathrm{1,2}}\left({E}_{1}\left({x}_{1}\right),{E}_{2}\left({x}_{2}\right)\right)\\ \times {f}_{1}\left({x}_{1}\right){f}_{2}\left({x}_{2}\right){f}_{3}\left({x}_{3}\right)\end{array}$$

The Vine copula structure consists of three parts called tree, branch, and node. Each layer of the tree consists of branches of two nodes, and two nodes of each branch are composed of Copula functions. Due to the non-uniqueness of the decomposition method of the joint density function, the vine copula is divided into three categories according to the different structures: C-vine, D-vine, and R-vine copula. Among them, C-vine and D-vine are special structures of vine copula. D-vine is decomposed by a specific rule, and the effect is not ideal in practical application. R-vine copula has a flexible structure, fewer restrictions, multiple nodes, and high accuracy in describing related structures. Therefore, the R-vine copula structure is adopted here [19].

For a random vector *X* obeying R-vine, ${X}_{D(e)}$ represents a sub-vector of *X* determined by the condition set $D(e)$. Then, the joint density function can be expressed as:

15 $$\begin{array}{c}f\left({x}_{1},{x}_{2},\dots ,{x}_{n}\right)=\left(\prod_{i=1}^{n-1} \prod_{e\in {E}_{\varnothing }} {c}_{i(e),k(e)\mid D(e)}\left({E}_{i(e)\mid D(e)}\left({x}_{i(e)}\mid {x}_{D(e)}\right),{E}_{k(e)\mid D(e)}\left({x}_{k(e)}\mid {x}_{D(e)}\right)\right)\right)\\ \times \left(\prod_{k=1}^{n} {f}_{k}\left({x}_{k}\right)\right)\end{array}$$

There is a conditional distribution function in the pair-copula, and the recursive equation of the conditional distribution function is as follows.

16 $$E(x\mid v)=\frac{\partial {C}_{x,{v}_{i}\mid {v}_{-i}}\left(E\left(x\mid {v}_{-i}\right),E\left({v}_{i}\mid {v}_{-i}\right)\right)}{\partial F\left({v}_{i}\mid {v}_{-i}\right)}$$

In Eq. (16), ${B}_{x,{v}_{i}\mid {v}_{-i}}$ is a pair-copula. ${v}_{i}$ is an arbitrary component of *v*. ${v}_{-i}$ is the other component of *v* except for ${v}_{i}$.

#### R-vine copula modeling

Suppose there is a sample $\left\{{u}_{mi}\right\},m=\mathrm{1,2},\cdots N,i=\mathrm{1,2},\cdots n$. *N* represents each sample number, and *n* represents the sample number. All marginal distributions are assumed to be uniform [20].

The first step is to calculate the empirical Kendall’s $\sigma$ value ${\widehat{\sigma }}_{i,k}$ for every two samples $\left({u}_{mi},{u}_{mk}\right),m=\mathrm{1,2},\cdots N$, 1 $\le i<k\le n$.

The second step is to select the maximum spanning tree based on absolute empirical Kendall’s $\sigma$ value, as shown in Eq. (17).

17 $${T}_{1}=\underset{T=(N,E) \, }{\mathrm{arg}}\sum_{e\in E} \left|{\widehat{\sigma }}_{i(e),k(e)}\right|$$

The third step is to select a copula ${c}_{i(e),k(e)}$ for each edge $e\in {E}_{1}$ to estimate the parameters ${\varnothing }_{i(e),k(e)}$. For $m=\mathrm{1,2},\cdots N$, conditions copula ${B}_{i(e),k(e)}\left({u}_{mi(e)}\mid {u}_{mk(e)}\right)$ and ${B}_{k(e)\mid i(e)}\left({u}_{mk(e)}\mid {u}_{mi(e)}\right)$ are calculated.

The fourth step is about $i=2,\cdots n-1$. For all conditional paired variables $\{\varnothing (e),i(e)\mid D(e)\}$, the empirical Kendall’s $\sigma$ value ${\widehat{\sigma }}_{i(e),k(e)\mid D(e)}$ is calculated according to the condition. The condition is as follows.

18 $$\left({B}_{i(e)\mid D(e)}\left({u}_{m,i(e)}\mid {u}_{m,D(e)}\right),{B}_{k(e)\mid D(e)}\left({u}_{m,k(e)}\mid {u}_{m,D(e)}\right)\right)m=\mathrm{1,2},\dots ,N$$

In ${E}_{p}$, the maximum spanning tree is selected according to Kendall’s $\sigma$ value [21].

19 $${T}_{\varnothing }=\underset{T=(N,E)E{\epsilon E}_{p} \, }{\mathrm{argmax}}\sum_{e\in E} \left|{\widehat{\sigma }}_{\varnothing (e),i(e)\mid D(e)}\right|$$

For each $e\in {E}_{\varnothing }$, a copula ${B}_{i\left(e\right),k\left(e\right); D(e)}$ is selected to estimate the corresponding parameters ${\varnothing }_{i\left(e\right),k\left(e\right); D(e)}$. For $m=\mathrm{1,2},\cdots N$, ${B}_{i\left(e\right),k\left(e\right); D(e)},{B}_{k\left(e\right),i\left(e\right); D(e)}$ is used to calculate ${B}_{i(e)\mid k(e)\cup D(e)}\left({u}_{m,k(e)}\mid {u}_{m,k(e)\cup D(e)}\right)$ and ${B}_{k(e)\mid i(e)\cup D(e)}\left({u}_{m,k(e)}\mid {u}_{m,i(e)\cup D(e)}\right)$.

The AIC and BIC principles are generally used to select Pair-Copula. *AIC* and *BIC* can be expressed as:

20 $$\begin{array}{c}AIC=-2lnL+2k\end{array}$$

21 $$\mathrm{BIC}=-2\mathrm{ln}L+k\mathrm{ln}(n)$$

### Factors affecting ExRates

**Fig. 2:**
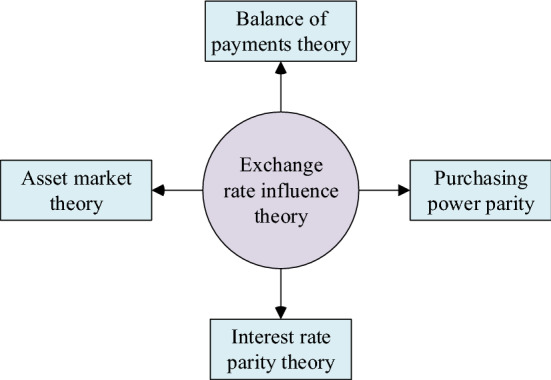
Theories related to the impact of ExRate

The ExRate refers to the rate of exchange between two currencies. It can also be regarded as the value of one country's currency against another. The ExRate issue has always been one of the core issues in international finance. Many factors affect a country’s currency ExRate, including trade balances, price levels, money supply, Rate of Interest (ROI) levels, and foreign exchange reserves [22]. There are four main theories affecting ExRates today, as displayed in Fig. 2.

In Fig. 2, theories related to ExRate impact include the balance of payments theory, purchasing power parity theory, interest rate parity theory, and asset market theory. The balance of payments theory holds that the balance of payments determines the foreign exchange supply and demand, which determines the ExRates, so the ExRate is ultimately determined by various factors affecting the balance of payments. If the balance of payments of a country is in surplus, the demand for the local currency is greater than the supply in the foreign exchange market, which will promote the appreciation of the local currency, and the same goes for the deficit [23].

Interest rate parity theory analyzes the relationship between ExRate and interest rate from the perspective of the financial market. It describes an equilibrium state of ExRate. According to the interest rate parity theory, the expected returns of all monetary assets in different countries should be the same, otherwise, there will be arbitrage space. The market's arbitrage mechanism eventually converges the expected returns on similar assets across countries. The ExRate depends on the comparison of interest rates between the two countries, and the equilibrium ExRate is formed through the foreign exchange trading behavior caused by international selling arbitrage.

The core of the purchasing power parity theory is the ExRate of the two currencies, which is determined by the domestic purchasing power ratio of the two currencies. The ExRate of the two currencies is determined by the rise and fall of the price level of the two countries. The real ExRate of a country remains unchanged, and the purchasing power ratio is the purchasing power parity [24]. Asset market theory believes that the imbalance in the asset market will cause the ExRate to change. Changes in ExRates push asset markets from unbalanced to balanced. The ExRate is the relative price of two currencies when the asset market is in equilibrium [25].

**Fig. 3:**
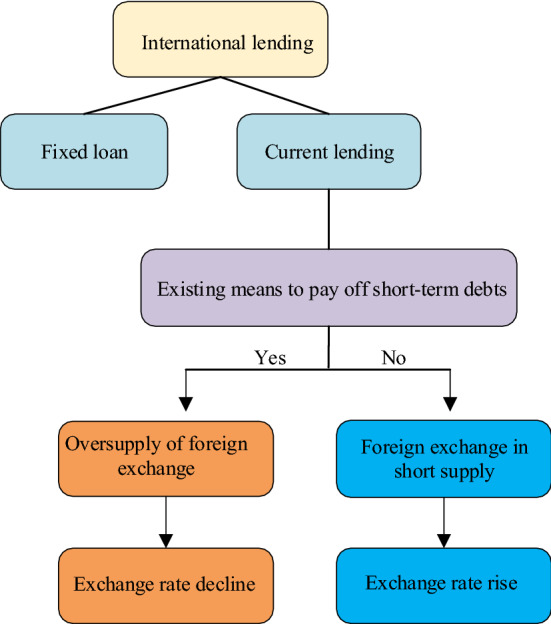
The theoretical mechanism of the BoP

The balance of payments theory, also known as the international lending theory, is a modern form of international lending theory. International lending refers to the comprehensive situation of a country's external claims and debts at a certain date, and this is a static concept. The balance of payments stands for the general situation of all the external economic transactions of a country in a certain period. The balance of payments is a dynamic concept, which has a wider scope than the original international lending economic transactions and is more in line with modern transaction forms. This theory emphasizes the effect of changes in the BoP on changes in currency ExRates. It believes that the SND of foreign exchange is generated by the lending behavior between two countries or regions [26]. Figure 3 displays its mechanism of action.

Figure 3 denotes that international lending includes two common methods: fixed and current. Only liquid lending goes into the disbursement phase. The impact on foreign ExRates is manifested in two aspects. The ExRate falls when short-term debt cannot be serviced with existing means of payment. The ExRate will rise when debts can be paid in cash. The main accounts for the balance of payments are the current account, capital and financial account, reserve asset account, net error, and omission account. Factors leading to measurement errors in the international balance of payments account mainly include cyclical factors, income factors, structural factors, monetary factors, and policy imbalance factors [27].

**Fig. 4:**
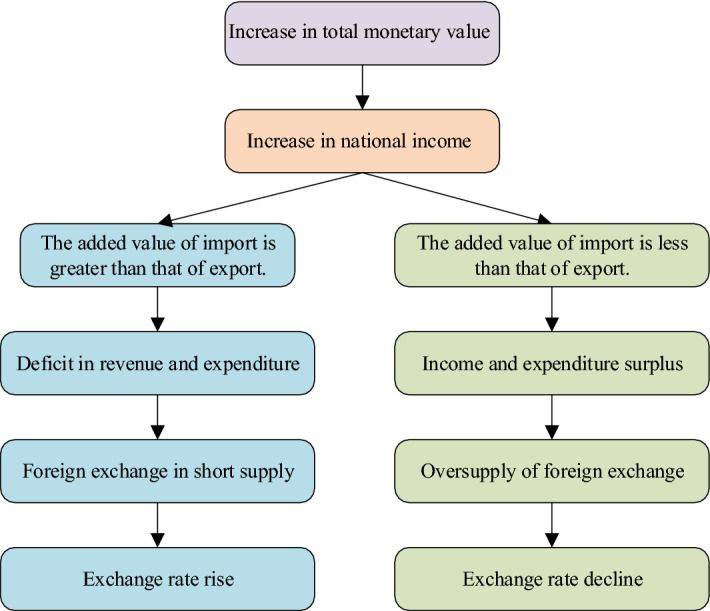
The function logic of modern BoP theory

The main reason that affects ExRate fluctuations is the changes in the total currency value involved in the various exchange, distribution, and other activities, which are changes in the BoP account funds [28]. Figure 4 displays the function logic of modern BoP theory.

National income increases when the total value of money increases. The BoP will be in deficit, foreign exchange will be in short supply, and the ExRate will rise when the added value of a country’s imports is more significant than the added value of its exports. There will be a surplus in the BoP, foreign exchange will oversupply, and the ExRate will decline when the added value of imports is less than the added value of exports.

**Fig. 5:**
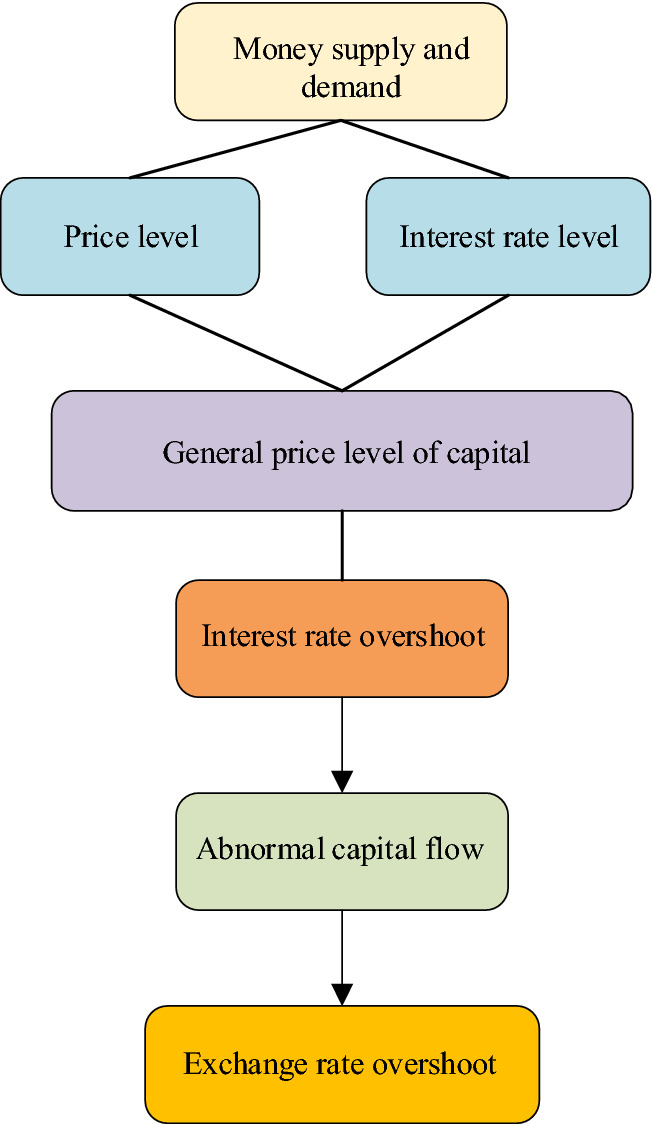
The theoretical function logic of the asset market

Asset currency theory mainly studies the logic of the role of money SND on ExRates. The specific logical relationship is shown in Fig. 5.

In Fig. 5, the situation of money supply and demand affects the price level and interest rate, as well as the general price level of capital in the capital market. The fluctuation of money supply and demand makes the interest rate change and inevitably affects the fluctuation of the ExRate price. This theory assumes that domestic and foreign non-monetary current assets are not completely substitutable, and the behavior of asset holders to adjust their monetary assets and portfolio of marketable securities according to risks and expectations will affect the situation of foreign exchange supply and demand, and then affect the change of ExRate. Thereupon, the ExRate level can only be determined when the bond market, foreign exchange market, and money market reach general equilibrium. The theory is a dynamic model that combines short-term flow and long-term stock, taking into account accumulated asset rate, current account balance, expectation and risk return, asset price, wealth level, and other factors.

### The mechanism of the trade war between China and America war on the RMB ExRate

**Fig. 6:**
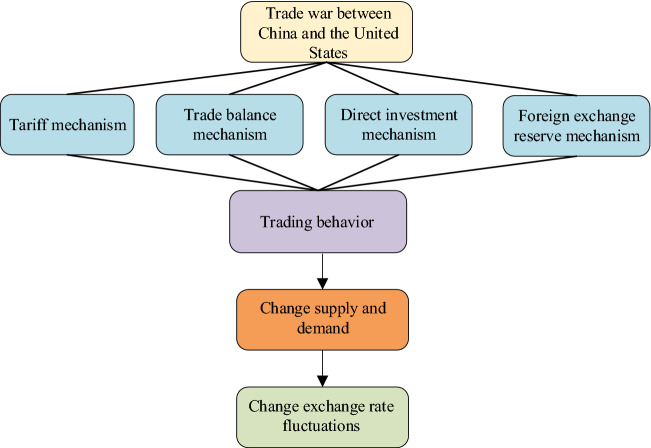
The impact mechanism of the trade war between China and America on the RMB ExRate

The mechanism of the impact of the trade war between China and America on the RMB ExRate is shown in Fig. 6.

In Fig. 6, the tariff, trade balance, direct investment, and foreign exchange reserve mechanisms play an important role in the entire transaction behavior. They change the SND situation, so the ExRate fluctuates. Tariffs will affect the RMB ExRate through inflation, trade scale, terms of trade, and employment rates [29]. ExRate features are mainly reflected in the trade balance mechanism and direct investment mechanism. The trade balance mechanism is manifested in the trade war, the US imposed tariffs and other measures to restrict China's exports to the US, thus reducing China's trade surplus with the US, while China also responded to the trade war initiated by the US through anti-dumping, tariff increase and other measures [30, 31]. The direct investment mechanism is manifested in the following aspects, the US takes restrictions on China's direct investment, which limits the outflow of Chinese capital, directly affects the flow of Chinese capital to the US market, and causes the change of currency demand in the foreign exchange market. Adequate foreign exchange reserves play an important role in the development of China's economy and have a positive significance for the internationalization of RMB. They also reflect the confidence and control power of the People's Bank of China in stabilizing the RMB ExRate. However, as USD assets account for a large proportion of China's foreign exchange reserve assets, a large fluctuation of the ExRate between the USD and RMB may lead to a large shrinkage of China's foreign exchange reserves.

### Data sources and research objects

In the context of the trade war between China and America, the selected research object is the daily closing price of the currency ExRates of the “Association of Southeast Asian Nations (ASEAN)” against the RMB. The R-vine copula model is used to study the interdependent structure of the ExRate market. The data comes from the official website of the Board of Governors of the Federal Reserve System (Fed), the Commerce Data Center of the Ministry of Commerce of the People’s Republic, and the official website of the Customs of the people’s Republic of China, and the Foreign Exchange Reserve Network. Relevant software includes R language and MATLAB.

The daily closing price series of the ExRate is non-stationary, and the logarithmic difference is multiplied by 100 as the return series instead of the original data for analysis and research. For the convenience of expression, the relevant letter symbols are used to replace the series of ExRates of the relevant ASEAN countries against the RMB currency ExRate. The details are revealed in Table 1.

**Table 1:** Relevant ASEAN countries

Country	Currency name	English abbreviation
Brunei	Brunei Dollar	BND
Indonesia	Indonesian Rupiah	IDR
Myanmar	Myanmar Kyat	MMK
Malaysia	Malaysian Ringgit	MYR
Singapore	Singapore Dollar	SGD
Thailand	Thai Baht	THB

## Results and discussion

### Changes in RMB ExRate under the trade war between China and America

The ExRate of China’s RMB at the end of each month from 2020 to 2021 is sorted out from the Foreign Exchange Reserve Network, as exhibited in Table 2.

**Table 2:** RMB ExRate from 2020 to 2021

Time	2020	2021
End of January	6.9109	6.4642
End of February	7.0192	6.468
End of March	7.0923	6.5652
End of April	7.0661	6.4672
End of May	7.1515	6.3637
End of June	7.0667	6.4561
End of July	6.9839	6.4623
End of August	6.8571	6.4684
End of September	6.8126	6.4615
End of October	6.6896	6.3906
End of November	6.5943	6.3717
End of December	6.5176	6.3689

**Fig. 7:**
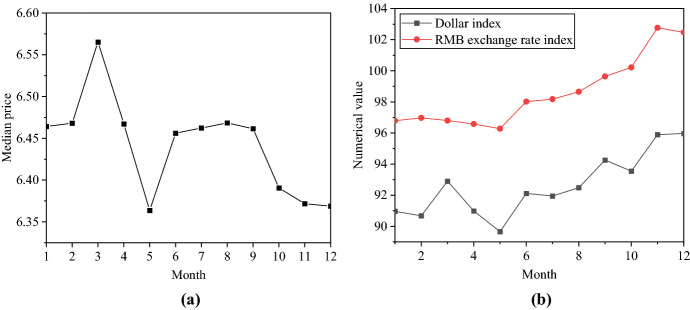
The ExRate trend of RMB against the USD. **a** The central parity rate of RMB against the USD; **b** The USD index and the RMB ExRate index

The changing trend of the central parity rate of RMB against the USD in 2021 and the relationship between the USD index and the RMB ExRate index are analyzed. Figure 7 shows the details.

In Fig. 7, the central parity rate of RMB against the USD changed from 6.4642 to 6.3689 from January 31, 2021, to December 31, 2021. In 2021, the movement of the RMB against the USD was roughly divided into two stages. The first stage is from December 31, 2020, to September 30, 2021. There is a significant negative correlation between the ExRate of RMB against the USD and the USD index. During this period, the RMB appreciated against the USD when the USD index fell. The second stage is from September 30, 2021, to the end of 2021. The RMB ExRate against the USD and the USD index shows a rising pattern. The USD index rose from 94.2638 to 95.9701 from September 30, 2021, to December 31, 2021. During the same period, the central parity rate of RMB against the USD changed from 6.4854 to 6.3757. The most important reason for the significant rise in the USD index was that the inflation rate in the US had hit record highs under the stimulus of expansionary policies. The Fed had to start normalizing monetary policy. US short-term and long-term ROI rose, pushing the dollar index up. The ExRate of RMB against the USD continued to appreciate in the context of the upward trend of the USD index.

### Analysis of the impact of the trade war between China and America on the RMB ExRate

#### The impact of tariffs on the RMB ExRate

**Fig. 8:**
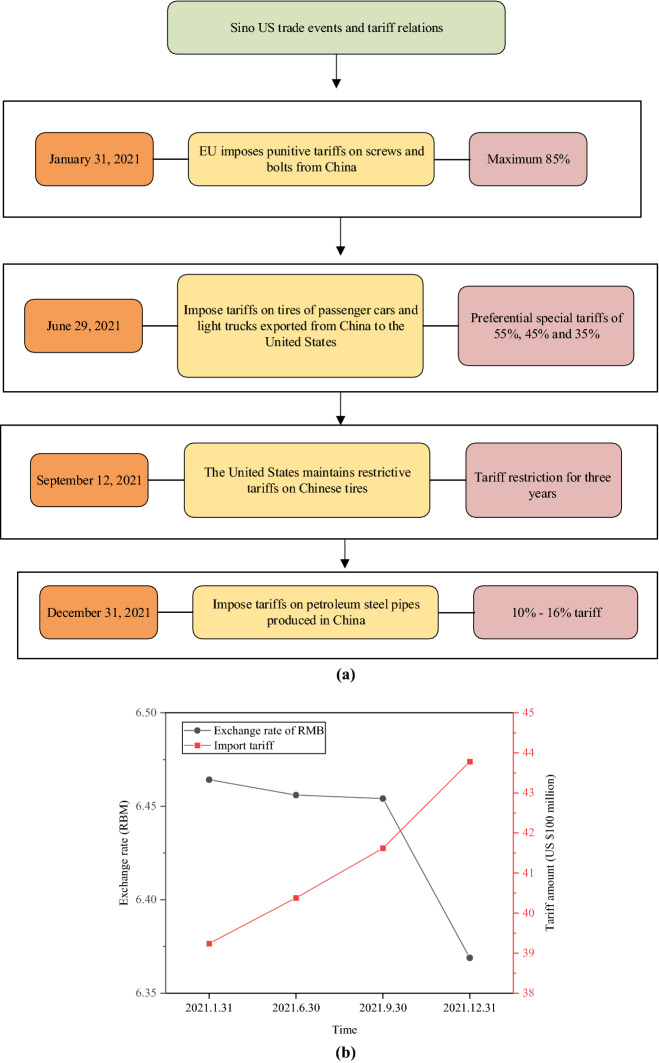
The impact of additional tariffs on the RMB ExRate. **a** Changes in the trade war between China and America and tariffs; **b** The relationship between tariffs and the RMB ExRate

The relationship between the trade war between China and America and tariffs in 2021 and the impact on the RMB ExRate are analyzed, as plotted in Fig. 8.

The relationship between the trade war between China and America and tariff changes is presented in Fig. 8a, and the relationship between tariffs and the RMB ExRate is shown in Fig. 8b. On January 31, 2021, the European Union imposed a punitive tariff of up to 85% on screws and bolts from China. On June 29, 2021, the United States International Trade Commission (ITC) imposed 55%, 45%, and 35% special tariffs because Chinese tires disrupted the US market. On September 12, 2021, the restrictive tariffs on Chinese tires remained in place for three years. On December 31, 2021, the ITC agreed to impose tariffs of about 10%-16% on Chinese-made petroleum steel pipes. There is a relation between the RMB ExRate and Chinese tariffs. The RMB ExRate is trending down when tariffs are trending up.

#### The impact of the trade war between China and America surplus on the RMB ExRate

**Fig. 9:**
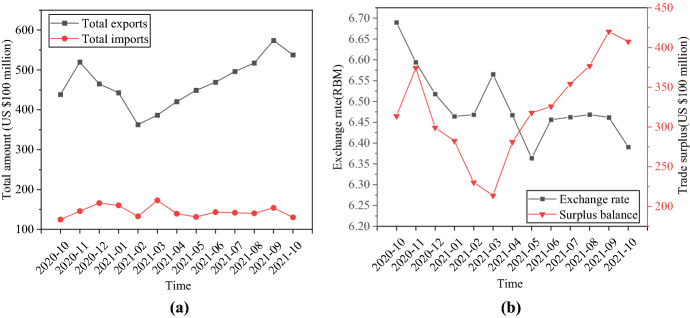
Impact of Trade war between China and America surplus on the RMB ExRate. **a** Comparison of China’s import and export volume to the US; **b** The relationship between China’s trade balance with the US and the RMB ExRate

The most direct purpose of the trade war is to reduce China’s trade surplus with the US to balance the international trade between China and the US. Figure 9 reveals the impact of narrowing the Trade war between China and America surplus on the RMB ExRate.

Figure 9 indicates that China’s exports and imports have always been surplus. In 2020, China’s trade surplus with the US showed a downward trend. China implemented trade war countermeasures. In May 2021, China’s trade surplus with the United States rose again. China’s trade surplus continues to extend when China and the United States have bilateral trade exchanges based on the BoP theory. The scale of US exports to China is smaller than the scale of its imports to China. Market demand for Chinese currency will also increase. Foreign exchange inflows. Chinese currency appreciates. Therefore, theoretically, if China’s trade surplus increases, the value of the RMB will increase without considering monetary policy. If China’s trade surplus becomes small, the value of the RMB gets low.

#### The impact of China's direct investment in the US on the RMB ExRate

**Fig. 10:**
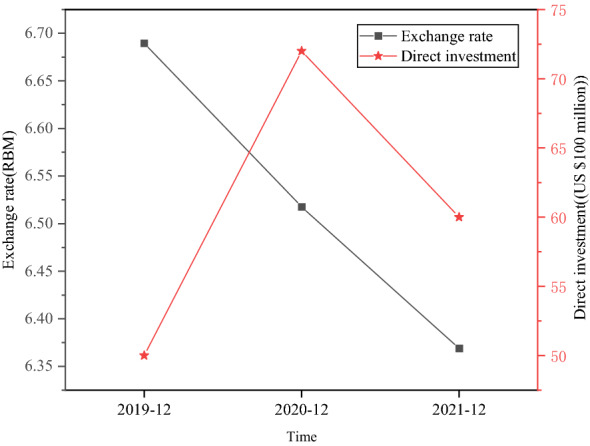
The impact of China’s direct investment in the US on the RMB ExRate

The impact of China’s direct investment in the US on the RMB ExRate in the past three years is analyzed, as shown in Fig. 10.

China’s direct investment in the US can bring the agglomeration effect of RMB currency settlement and the substitution effect of the USD currency. China can promote frequent settlement and exchange between RMB currency and USD currency and increase and expand the frequency and scope of RMB foreign settlement through direct investment in the US and its promotion of import and export trade with the US. The trade war between China and America has restricted China’s direct investment in the US and the outflow of Chinese capital, putting pressure on the RMB to depreciate.

#### The impact of stabilizing the scale of foreign exchange reserves on the RMB ExRate

**Fig. 11:**
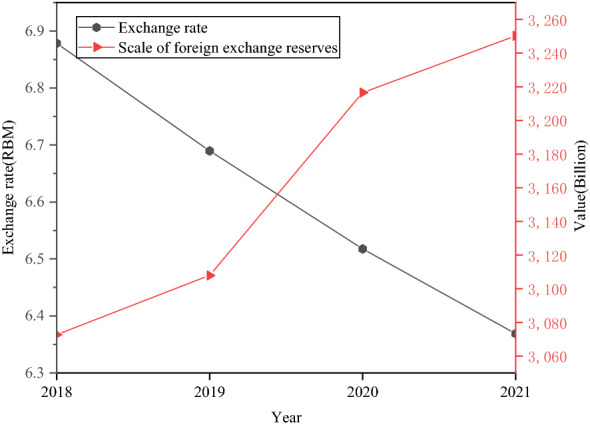
The impact of stable foreign exchange reserves on the RMB ExRate

The impact of stabilizing the scale of foreign exchange reserves on the RMB ExRate is analyzed, as expressed in Fig. 11.

Foreign exchange reserves play a central role in balancing domestic and international payments and stabilizing ExRates. Figure 11 shows China’s foreign exchange reserves have remained above US$3 trillion in the past four years. Stable foreign exchange reserves can stabilize the RMB ExRate. The scale of foreign exchange reserves is reduced, and the RMB ExRate is appropriately raised.

### R-vine copula model analysis

#### Fluctuations in the ExRate yield sequence of ASEAN countries

**Fig. 12:**
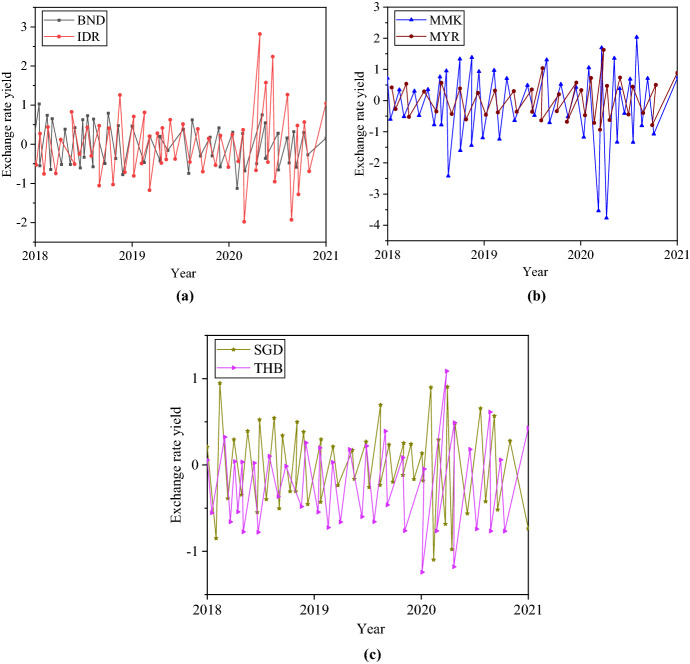
Sequence of ExRate yields of ASEAN countries. **a** BND and IDR ExRate yield; **b** MMK and MYR ExRate yield; **c** SGD and THB ExRate yield

The series fluctuations of the ExRate yields of ASEAN from 2018 to 2021 in different countries are analyzed. The results are demonstrated in Fig. 12.

Figure 12 shows apparent volatility clustering coefficients, persistence coefficients, and jumping reactions in the return series. Each yield series fluctuate significantly after 2020. This is mainly because of the outbreak of the new crown epidemic. The trade war between China and America has increasingly affected economic and trade cooperation between China and ASEAN. The ExRate risk between the two places has increased, and the ExRate yield sequence has fluctuated.

#### Market dependence coefficient analysis of currency ExRate of ASEAN countries

**Fig. 13:**
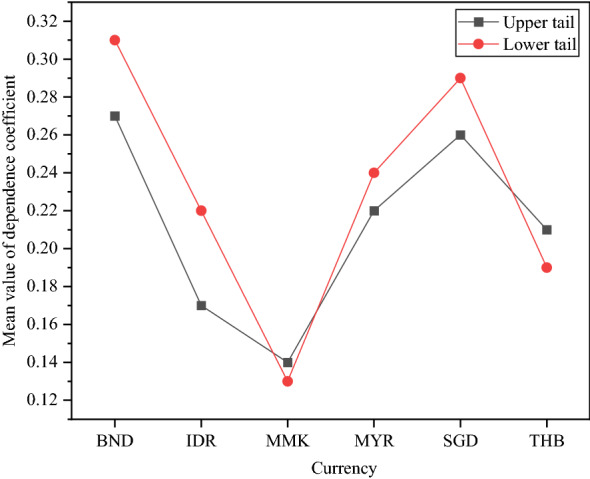
The mean value of the upper and lower tail dependence coefficients of the currency ExRates of ASEAN countries and other countries

The mean value of the upper and lower tail dependence coefficients of the currency ExRate markets of ASEAN is analyzed. Figure 13 reveals the results.

The lower tail dependency coefficient for market tail dependencies is slightly larger than the upper tail dependency coefficient. Only the Myanmar kyat and the Thai baht have lower-tail dependency coefficients than upper-tail dependency coefficients. For most ASEAN countries, the value of the lower tail dependency coefficient is larger than the upper tail dependency coefficient. This shows that bad news significantly impacts the currency ExRate market. The mean values of the external tail dependence coefficients of the ExRates of BND and SGD are both high. The results imply that economically developed countries occupy a dominant position in the ASEAN ExRate market.

The results of the overall dependence coefficient of currency ExRate market of ASEAN by using the R-vine copula model are outlined in Table 3.

**Table 3:** The dependence coefficient of currency ExRate market of ASEAN

Currency	BND	IDR	MMK	MYR	SGD	THB
BND	1	0.3754	0.2192	0.4245	0.8999	0.5679
IDR	0.3754	1	0.2516	0.5812	0.3662	0.3052
MMK	0.2192	0.2516	1	0.2701	0.1917	0.2162
MYR	0.4245	0.5812	0.2701	1	0.4229	0.3236
SGD	0.8999	0.3662	0.1917	0.4229	1	0.6033
THB	0.5679	0.3052	0.2162	0.3236	0.6033	1

Table 3 presents that the dependence coefficient of the currency ExRate between BND and SGD is 0.8999. This is mainly due to the frequent trade and capital exchanges between Singapore and Brunei, which promoted the increase of the linkage coefficient of the ExRate market. The dependence coefficient between IDR and MYR is the second, with 0.5812. The dependence coefficient is closely related to the ExRate market.

#### Estimation results of R-vine copula parameters in currency ExRate markets of ASEAN countries

**Fig. 14:**
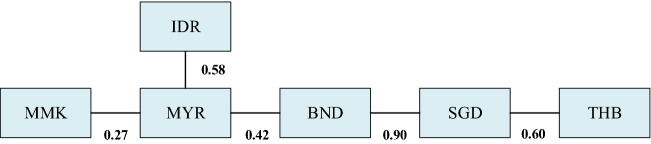
The R-vine dependent structure of the currency ExRate market of ASEAN

The R-vine dependent structure of the currency ExRate market of ASEAN and the analysis of estimation results of the R-vine copula parameter are indicated in Fig. 14 and Table 4.

**Table 4:** The estimation results of the R-vine copula parameter

Tree structure	Copula	Parameter 1	Parameter 2	$\tau$	λ_U_	$\lambda_{L}$
1, 2	T-copula	0.26	6.63	0.27	0.06	0.06
2, 3	T-copula	0.57	9.47	0.58	0.13	0.13
2, 4	T-copula	0.41	7.16	0.42	0.12	0.12
4, 5	T-copula	0.98	1.98	0.90	0.92	0.92
5, 6	T-copula	0.59	6.22	0.40	0.21	0.21

In Fig. 14, the ExRate market of MYR is located at the central point of the volatility transmission of the ExRate market of ASEAN and is closely connected with other ExRate markets. ExRate markets of BND, SGD, and THB become a branch, and the ASEAN market has an obvious geographical aggregation effect. In Table 4, MMK, MYR, IDR, BND, SGD, and THB are respectively represented by 1,2,3,4,5,6. Except for MYR and IDR, the dependence coefficient of BND and SGD ExRates is less than 0.5, others are above 0.5.

## Conclusion

This paper first introduces the Copula function theory, the correlation measure of the copula function, R-vine copula correlation theory, and further constructs the R-vine copula model. The influencing factors of ExRate and the mechanism of the trade war between China and America on the RMB ExRate are studied. Besides, the R-vine copula model empirically analyzes the RMB ExRate market. The impact of the trade war between China and America on the RMB ExRate is analyzed in terms of tariffs, trade war between China and America balance, China’s direct investment in the US, and the scale of stable foreign exchange reserves. The R-vine copula model is adopted to explore the fluctuation of ExRate return series in ASEAN countries, and the market dependence coefficient of currency ExRates in ASEAN countries and the estimation results of R-vine copula parameters are analyzed. The following conclusions are drawn.

Although the trade war between China and America has persisted, the RMB ExRate has been on a slow downward trend. This shows that the RMB has always been in a state of appreciation and can maintain steady development within a range as a whole. There is an upward trend in the USD and RMB ExRate indexes. In the analysis of the impact of the trade war between China and America on the RMB ExRate, there is a linkage between the RMB ExRate and Chinese tariffs. The RMB ExRate is trending down when tariffs are trending up. There is a positive correlation between the value of the RMB and trade surplus, regardless of monetary policy. If China’s trade surplus is large, the value of the RMB’s currency will rise. Stable foreign exchange reserves can stabilize the RMB ExRate. The scale of foreign exchange reserves is reduced, and the RMB ExRate is appropriately raised. It is concluded that the economic and trade cooperation between China and ASEAN has become increasingly frequent under the influence of the trade war between China and America through the R-vine copula model analysis. The ExRate risk further increases, and the ExRate yield sequence fluctuates significantly. The lower tail dependency coefficient for market tail dependencies is slightly larger than the upper tail dependency coefficient. The impact of bad news on the currency ExRate market is clear. In the context of the current trade war between China and America intensifying, the sharp fluctuations in the RMB ExRate caused by the trade war between China and America have promoted the transformation of economic growth. The shift from commodity export to capital export is realized. The reasons for RMB ExRate changes need to be explored. It is necessary to enhance China’s economic strength in the future continuously. Promoting the process of RMB internationalization is the decisive factor in the fluctuation of the RMB ExRate. It is suggested that the reform of the ExRate mechanism should continue to be deepened, the ExRate should be more flexible, and the reform of the RMB ExRate mechanism should be deepened, thereby forming a floating ExRate mechanism based on market supply and timely management. Moreover, currency swaps among ASEAN countries need to be strengthened to promote the internationalization of RMB.
